# Development and validation of the Individual Potentials Questionnaire (IP-Q)

**DOI:** 10.1038/s41598-025-90868-6

**Published:** 2025-03-28

**Authors:** Abdulwali Sabo, Garry Kuan, Sarimah Abdullah, Hue San Kuay, Yee Cheng Kueh

**Affiliations:** 1 Biostatistics and Research Methodology Unit, School of Medical Sciences, Universiti Sains Malaysia, Kubang Kerian, Kelantan Malaysia; 2 Department of Public and Environmental Health, Faculty of Basic Medical Sciences, Federal University Dutse, Dutse, Jigawa State Nigeria; 3 Exercise and Sports Science Programme, School of Health Sciences, Universiti Sains Malaysia, Kubang Kerian, Kelantan Malaysia; 4 Department of Psychiatry, School of Medical Sciences, Universiti Sains Malaysia, Kubang Kerian, Kelantan Malaysia

**Keywords:** Demands, Life, Holistic, Health, Reliability, Construct

## Abstract

Individual potential has recently been acknowledged by the holistic health model as being essential to successfully addressing life’s demands, both now and in the future. The study employed a cross-sectional survey among Nigerian university undergraduate students, using a convenience sampling method, to assess their subjective individual potential. The study investigated the psychometric properties of the newly developed Individual Potentials Questionnaire (IP-Q). The study involved a total of 730 participants (EFA = 300 and CFA = 430). The I-CVIs and S-CVIs fall within the range of 0.83 to 1, and the I-FVIs and S-FVIs are 1. Two factors (biologically given potential and personally acquired potential) emerged in the EFA analysis, with all 14 items retained due to satisfactory factor loadings (above 0.50) and KMO = 0.905 (*p*-value < 0.001). The final CFA model fit indices were: CFI = 0.984, TLI = 0.980, SRMR = 0.034, RMSEA = 0.041, and RMSEA *p*-value = 0.880. Furthermore, the ICCs for the test–retest are 0.976 (biologically given potential) and 0.953 (personally acquired potential). The results show that the newly developed IP-Q has adequate construct validity and able to assess subjective individual potential.

## Introduction

Most individuals strive to maintain control over their lives and future trajectory while maintaining close relationships with family and friends within their social circles. However, these relationships naturally evolve as people age^1^. Additionally, newborns are entirely dependent on caregivers, a reliance that intensifies during illness or the frailty of old age. As a result, individuals must continually adapt to age- and culturally specific demands and challenges throughout each stage of life^1^. This adaptability highlights that an individual’s ability to meet short-, medium-, and long-term needs serves as a reflection of their overall health status^2^. The concept of “potential” effectively encompasses all prospective abilities required to address life’s needs^1,3^.

The Meikirch model of holistic health emphasizes the importance of individual potential in effectively meeting life’s demands, both in the short and long term^1,3^. This model defines individual potential as comprising two dimensions: biologically given potentials and personally acquired potentials^1^. Biologically given potential refers to the innate biological basis of life, which begins at birth with a finite value determined by genetic factors and the quality of pregnancy and then declines over the course of life, eventually reaching zero at death. The personally acquired potential encompasses the total physiological, mental, and social resources a person accumulates throughout their life, which starts developing in utero and continues to grow as long as the individual actively seeks to foster their personal development and resides in a supportive and health-promoting social environment^1,3^.

Similarly, the Health Belief Model (HBM) is widely utilized to explain health-related behaviors by focusing on the perceptions and beliefs that drive decision-making^4,5^. Within the HBM, two key constructs are particularly relevant to individual potential: (1) perceived severity, which reflects an individual’s understanding of the seriousness of health issues, which fosters a proactive approach, encourages taking responsibility for well-being, and enhances resilience, and (2) perceived benefits**,** which reflect the belief in the effectiveness of actions, such as adopting healthy habits, which reinforce positive behaviors and instil confidence in personal growth^4^. Ultimately, the Meikirch model of holistic health and HBM offer valuable frameworks for understanding and fostering individual potential, particularly regarding health behaviors, personal development, and the perceptions that shape decision-making.

For instance, in a prior study, 87% of participants assessed their health as “excellent” or "very good." Notably, this percentage remain unchanged despite 36% reporting headaches, 37% experiencing backaches, 35% experiencing sleep difficulties, and 23% dealing with other significant conditions in the preceding four weeks^6^. This highlights the ability approach, which posits that individuals’ abilities to attain well-being depend on their actions and achievements, ultimately shaping the quality of life they can lead^7–9^. Consequently, human health systems should aim to enhance an individual’s functional abilities rather than focusing solely on end-state necessities such as health, pleasure, or fulfilment of desires^1^.

The concept of individual potential represents a multidimensional framework that includes inherent abilities, acquired skills, and the ability to adapt to life’s challenges^1,4^. Recognizing its critical role in personal development, well-being, and success, there is a clear need for an assessment scale to measure and evaluate individual potential systematically. The existing instruments are limited to assessing specific traits of individual potentials, such as the Self-Rated Health Questionnaire^10,11^, the Short Form Sense of Coherence Scale^12^, the Self-Efficacy for Exercise Scale^13^, and the Web-Based Headache Diagnosis Questionnaire^14^. None of these scales provide a holistic assessment of individual potential. To address this gap, the present study aims to develop the Individual Potentials Questionnaire (IP-Q), based on the two proposed constructs of individual potential, and evaluate its psychometric properties among undergraduate university students in Nigeria.

## Methods

### Study design and data collection

Researchers conducted a cross-sectional survey from April 2023 to June 2023, involving 730 undergraduate students from the College of Medicine and Allied Medical Sciences at Federal University Dutse, Nigeria (FUD). Convenience sampling is recognized as a more stringent approach for participant selection in research, but it may be appropriate for exploratory or preliminary investigations aimed at generating hypotheses or insights^15,16^. This method allows researchers to swiftly gather data, laying the groundwork for subsequent research activities^15^. Convenience sampling is applied for participant selection in this study because it is a straightforward and easily accessible approach that accurately represents the population from which the sample was drawn^17^, thereby establishing a strong basis for generalizability.

Students meeting the study’s inclusion criteria received the Google Form link for data collection due to its practicality, versatility, and cost-effectiveness. Researchers and surveyors commonly use Google Forms to effectively mitigate response bias^18^. The inclusion criteria included enrolment in Federal University Dutse (FUD) College of Medicine and Allied Medical Sciences; undergraduate status ranging from first to final year; registered students during the data collection period; and expressed consent to participate. We specifically selected participants from the College of Medicine and Allied Medical Sciences because they are presumed to be familiar with and comprehend the underlying concepts and constructs being assessed. Participants who have a certain level of understanding of a specific scale contribute to improving the scale’s construct validity, ensuring that it accurately measures the intended concepts^19^. In addition, all foreign students were excluded.

### Ethical approval

The Human Research Ethics Committee, Ministry of Health, Jigawa State, Nigeria [JGHREC/2023/151], and Universiti Sains Malaysia’s Human Research Ethics Committee [USM/JEPeM/22110695], granted ethical approval for the study. The participants were informed about the research aim and methods before signing the informed consent form. Written informed consent was obtained from each participant. The study conforms to the principles outlined in the Declaration of Helsinki.

### Scales’ item generation

Based on the Meikirch model^1,3^, the present study develops IP-Q, which posits two hypothetical constructs: biologically given potential and personally acquired potential. Expert input was sought from professionals in public health, psychometrics, health psychology, and questionnaire validation to refine the generated items. Additionally, in-depth interviews were conducted with twelve undergraduate students to gather further insights. Initially comprising 14 items, with six under biologically given potential and eight under personally acquired potential, the IP-Q was expanded to 56 items (14 × 4) by providing four alternative options for each original item. Subsequently, experts in pertinent fields evaluated these items to identify the most suitable 14, selecting one item from each set of four. The authors formulated the final set of 14 items through consensus. These items were evaluated using a four-point rating scale, ranging from 1 (none) to 4 (severe) for biologically given potential construct and from 1 (not at all) to 4 (very often) for personally acquired potential construct.

For the biologically given potential, the items were generated based on the HBM construct of perceived severity. Whereas, for the personally acquired potential, the items were generated based on the HBM construct of perceived benefits^4,5^. This construct suggests that even when individuals recognize their susceptibility to and the severity of a health threat, they are unlikely to adopt a recommended health action unless they perceive it as beneficial in mitigating the threat. Additional information was drawn from Antonovsky’s salutogenic model^20^, which emphasizes the sense of coherence as a key factor in effective stress management. This model describes a universal perspective in which individuals believe that the stimuli they encounter from both internal and external environments are structured, predictable, and understandable (comprehensibility); that resources are available to meet the demands posed by these stimuli (manageability); and that these demands are meaningful, worthwhile, and deserving of their effort and engagement (meaningfulness)^21,22^.

### Content validity

After item generation, six experts in health psychology, psychometrics, public health, and questionnaire development determined the content validity index (CVI). Through the use of a Google Form link, each expert assessed each item’s relevance to their specific construct. Next, we calculated the item content validity index (I-CVI) and scale content validity index (S-CVI) based on previously established standards^23–26^ to evaluate the CVI. For each item, the relevance rating was transformed to 0 (the item is not relevant or is somewhat relevant) or 1 (the item is quite relevant or highly relevant). We estimated the I-CVIs by calculating the proportion of the experts, giving the items a relevance rating of 1, and the I-CVIs for each construct were averaged to determine the S-CVI/Ave. In addition, we calculated the S-CVI/UA by averaging the proportion of scale items that achieved a 1 for relevance from all experts^24^. The I-CVIs for all 14 items ranged from 0.83 to 1. The S-CVIs/Ave were 1 for biologically given potential and 0.98 for personally acquired potential. The S-CVIs/UA were 1 for biologically given potential and 0.88 for personally acquired potential. As a result, these CVI values satisfied the required cut-up value of 0.83 (for six experts)^24^.

### Face validity

We also determined the face validity index (FVI) to assess the clarity and comprehension of the items. Ten undergraduate students from the targeted population evaluated each item for clarity and comprehension via a Google Form link. We estimated the item face validity index (I-FVI) and scale face validity index (S-FVI) based on the standard recommendations^27,28^. Each item was rated as either 1 (clear and understandable, or very clear and understandable) or 0 (not clear and understandable, or somewhat clear and understandable) based on relevance. We calculated the I-FVI by determining the proportion of students who assigned a relevance rating of 1 to the items. We computed the S-FVIs/Ave by averaging the I-FVIs for each construct on the IP-Q. Finally, we estimated the S-FVIs/UA by averaging the proportion of scale items that all students rated as 1 for relevance^28^. The I-FVIs for all 14 items are equal to 1. For the constructs, the S-FVIs/Ave and S-FVIs/UA were each rated as 1. As a result, these FVI values satisfied the required cut-off value of 0.83 (for 10 raters)^27^.

### Sample size estimation

The recommended minimum sample size for exploratory factor analysis (EFA) falls within the range of 100 to 250 individuals^29^. To account for potential missing values, we set the adjusted sample size at 286 by adding 30%. As a result, the EFA sample size is rounded to 300. Additionally, according to Tabachnick, Fidell^30^, a sample size of 300 is considered reasonable for EFA. Studies involving seven or fewer constructs in confirmatory factor analysis (CFA) should aim for a minimum sample size of 300^31^. Following this guideline, we maintained a sample size of 300 for the CFA phase. After incorporating a 30% correction for missing values, the final corrected sample size for CFA was 430.

### Statistical analysis

The data underwent pre-screening to identify erroneous data entries and missing values. Subsequently, EFA was carried out using Statistical Product and Service Solutions (SPSS) version 27 (IBM, Armonk, NY, USA). Following this, CFA was conducted using Mplus 8 to validate the EFA model. The researchers employed the MLR estimator during the CFA due to its robustness to non-normal data distributions^32^.

The EFA sample comprised 300 participants. To identify the principal contributing factors, the 14 items on the IP-Q underwent testing using principal axis factoring with Promax rotation selected. Researchers often select Promax rotation for EFA when they anticipate or have a theoretical rationale for correlated factors, as well as when seeking a more pragmatic and interpretable factor structure^30^. Furthermore, Promax rotation aids in better aligning the hypothesized model with established theories or expectations^30^. Upon identifying factors with eigenvalues surpassing one, those displaying factor loadings exceeding 0.40 were considered satisfactory and retained for subsequent CFA^26,33^. DeVon, Block^26^ also suggest that a Cronbach’s alpha value of 0.60 or higher indicates acceptable reliability for each factor.

CFA was applied to validate the EFA model with a sample size of 430 respondents. A standardized factor loading equal to or greater than 0.40 served as the criterion for retaining or removing an item in the current study^34,35^. According to Hair^36^, for sample sizes exceeding 250 and consisting of 12 items or more, the acceptable fit indices included: root mean square error of approximation (RMSEA) below 0.07; standardized root mean square residual (SRMR) below 0.08; and comparative fit index (CFI) or Tucker and Lewis index (TLI) exceeding 0.94. After considering adequate theoretical support, the model was revised based on the CFA modification index to enhance the model fit indices.

By computing composite reliability (CR) and average variance extracted (AVE), we were able to delve deeper into assessing the convergent validity of the IP-Q. Acceptable threshold values for CR and AVE were set at 0.70 and 0.50, respectively, or higher^26,33,37^. To evaluate discriminant validity, which investigates the extent to which one factor differs from another, correlations between factors were examined^33^. For discriminant validity, a correlation coefficient of 0.85 or less between two factors is considered adequate^33^. According to Fornell and Larcker^35^, confirmation of discriminant validity necessitates that the AVE of constructs exceeds the squared correlation coefficient, representing shared variance among variables. To assess test–retest reliability, a subset of 70 respondents completed the IP-Q twice within a seven-day period. A value exceeding 0.70 for the intra-class correlation coefficient (ICC) indicates satisfactory stability^38^.

## Results

### General characteristics

Table 1 presents the characteristics of participants in the EFA (n = 300) and CFA (n = 430) samples. We provided information regarding the students’ physical activity levels to offer insights into their lifestyles. Regular physical activity is reported to positively influence mental health, including alleviating symptoms of depression among university students^39^.

**Table 1:** General characteristics of the respondents in EFA and CFA (n = 730).

	EFA (300)		CFA (430)	
Variables	Mean (SD)	N (%)	Mean (SD)	n (%)
Age	21.15 (2.96)		22.38 (2.43)	
Frequency of exercise/week	4.05 (2.25)		3.44 (2.12)	
Duration of exercise (min)	46.22 (37.42)		46.16 (52.01)	
Gender				
Male		167 (55.7)		232 (54.0)
Female		133 (44.3)		198 (46.0)
Ethnicity				
Hausa		212 (70.7)		305 (70.9)
Yoruba		31 (10.3)		45 (10.5)
Igbo		11 (3.7)		6 (1.4)
Others		46 (15.3)		74 (17.2)
Field of study				
Medicine		131 (43.7)		230 (53.5)
Human anatomy		109 (36.3)		118 (27.4)
Human physiology		60 (20.0)		82 (19.1)
Study year				
Year 1		87 (29.0)		67 (15.6)
Year 2		66 (22.0)		63 (14.7)
Year 3		52 (17.3)		153 (35.6)
Year 4		95 (31.7)		147 (34.2)

N number, SD standard deviation.

### Exploratory factor analysis (EFA)

**Fig. 1:**
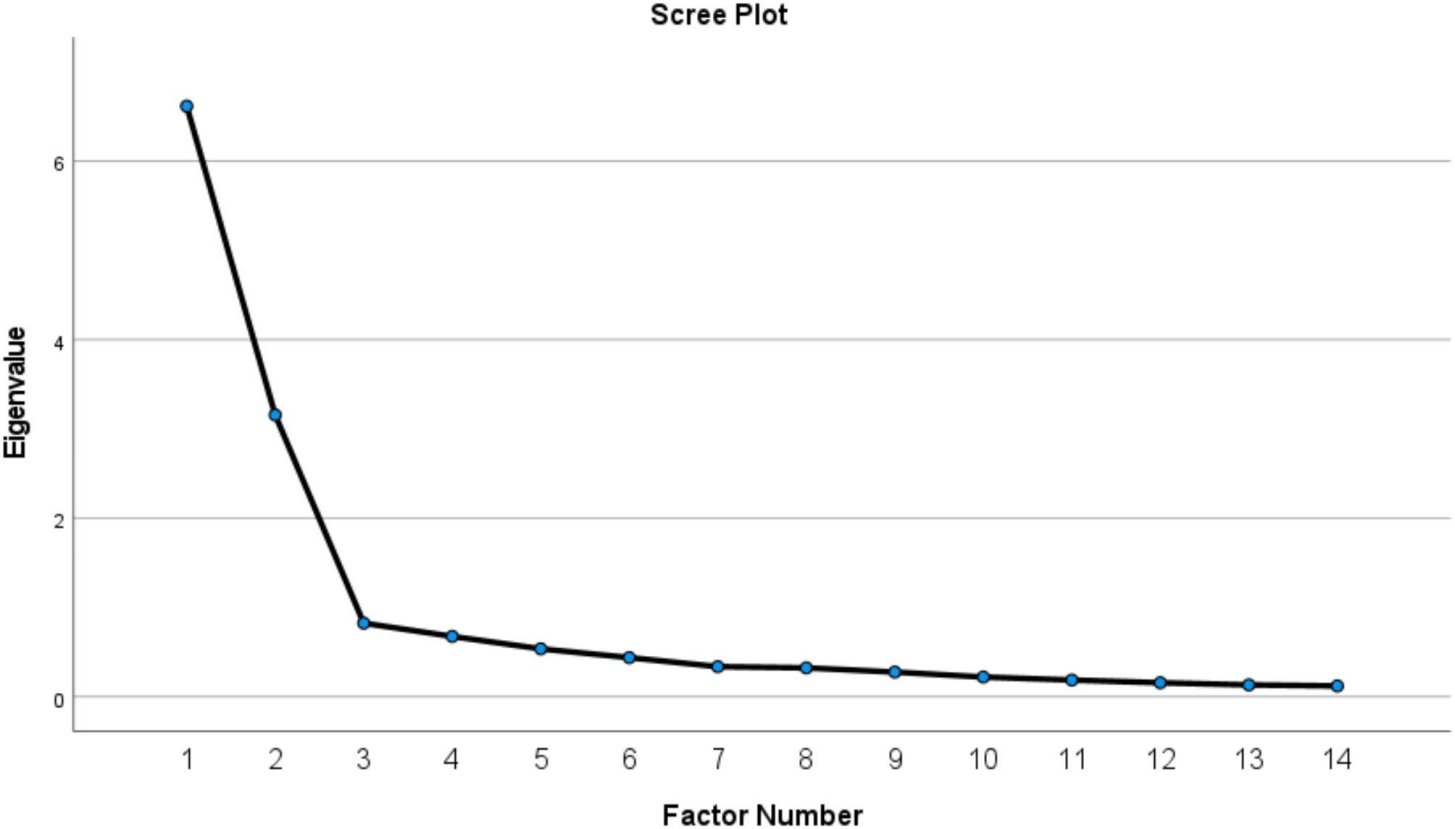
The scree plot of exploratory factor analysis (EFA).

The Bartlett’s sphericity test revealed a significant result (*p*-value < 0.001), and the estimated Kaiser–Meyer–Olkin (KMO) value of the EFA model of the original IP-Q with 14 items was 0.905. The model is thus considered to have adequate convergent validity. In the preliminary EFA model, we identified two factors with eigenvalues greater than 1 (Fig. 1) and satisfactory factor loadings for all items (Table 2). As a result, in the subsequent step, we set the number of factors at two in accordance with the initial hypothesised structure of the IP-Q. In order, to generate the two factors, we used Promax rotation and principal axis factoring. The two factors had a cumulative percentage of 69.81%, factor loadings > 0.40, no cross-loadings, and a factor correlation of − 0.361. Thus, the EFA indicated that no items needed to be removed.

**Table 2:** Items descriptive statistics, exploratory factor analysis, and reliability analysis (n = 300).

N	Item	Factor loading	Mean (SD)	Cronbach’s alpha
	Biologically given potential			0.928
1	Do you have any chronic health conditions that you developed as a child?	0.739	1.55 (0.85)	
2	During your early childhood, did you have any challenges because of your health condition?	0.726	1.55 (0.85)	
3	Do you have any health issues right now?	0.848	1.71 (0.92)	
4	Do you have any chronic conditions right now?	0.902	1.52 (0.86)	
5	Do you have any long-standing chronic conditions that have been present for at least six months?	0.901	1.57 (0.91)	
6	Do you have any chronic conditions that are limiting your daily activities?	0.835	1.49 (0.83)	
	Personally acquired potential			0.925
7	Do you believe that you can accomplish your life goals regardless of the circumstances?	0.701	3.23 (0.94)	
8	Do you feel that the changes in the past have made your situation unpleasant?	0.629	2.82 (1.03)	
9	When you are in an unfamiliar situation, does it affect your normal activities?	0.909	2.35 (0.87)	
10	How well do you solve your issues when faced with a challenge?	0.686	3.02 (0.84)	
11	Do you believe that your state of happiness may be affected by pain or health issues?	0.897	2.27 (1.03)	
12	How often do you experience regret over your past?	0.723	2.69 (0.92)	
13	How often do you feel bad about your future?	0.771	3.12 (0.98)	
14	How often do you feel in control of the conditions in your life?	0.933	2.77 (0.93)	

Principal axis factoring was applied, KMO = 0.905, Bartlett’s test of sphericity (*p* < 0.001), Total variance explained by two factors = 69.81%, Factor correlation =  − 0.361, item-total correlation = 0.731–0.848 (biologically given potential) and 0.607–0.885 (personally acquired potential).

### Confirmatory factor analysis (CFA)

**Fig. 2:**
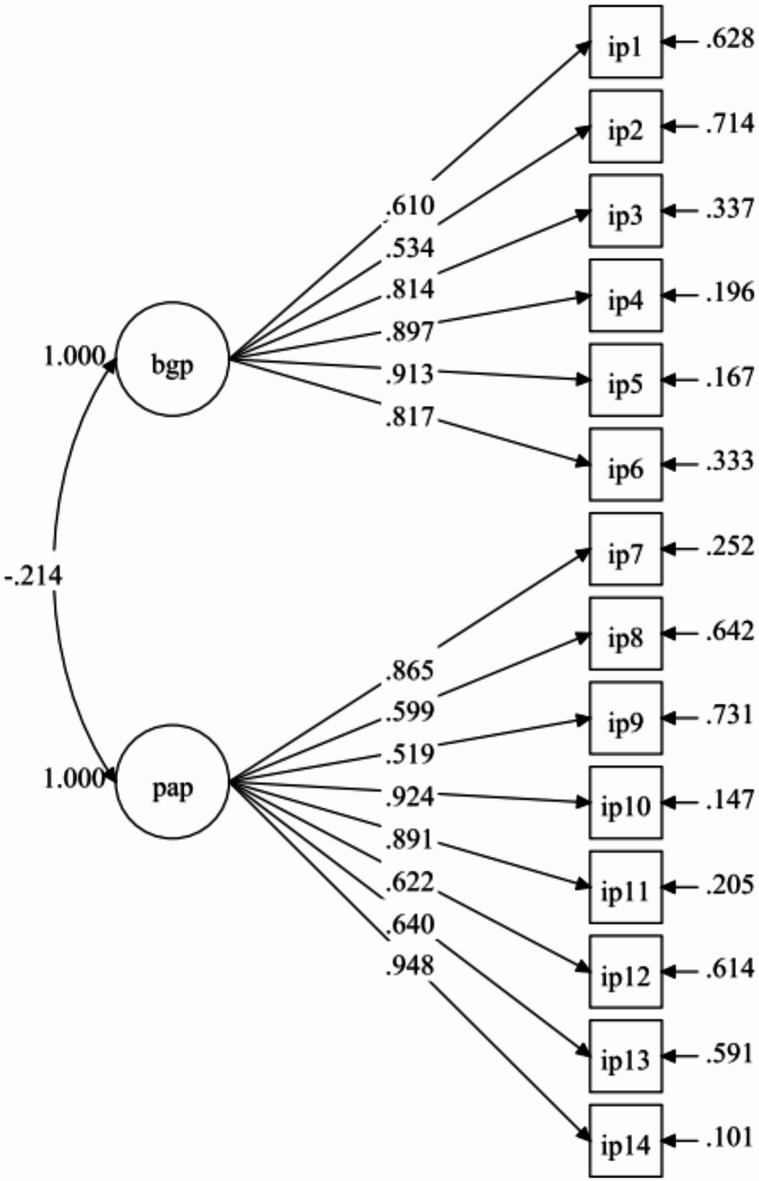
IP-Q measurement (Model-1).

**Fig. 3:**
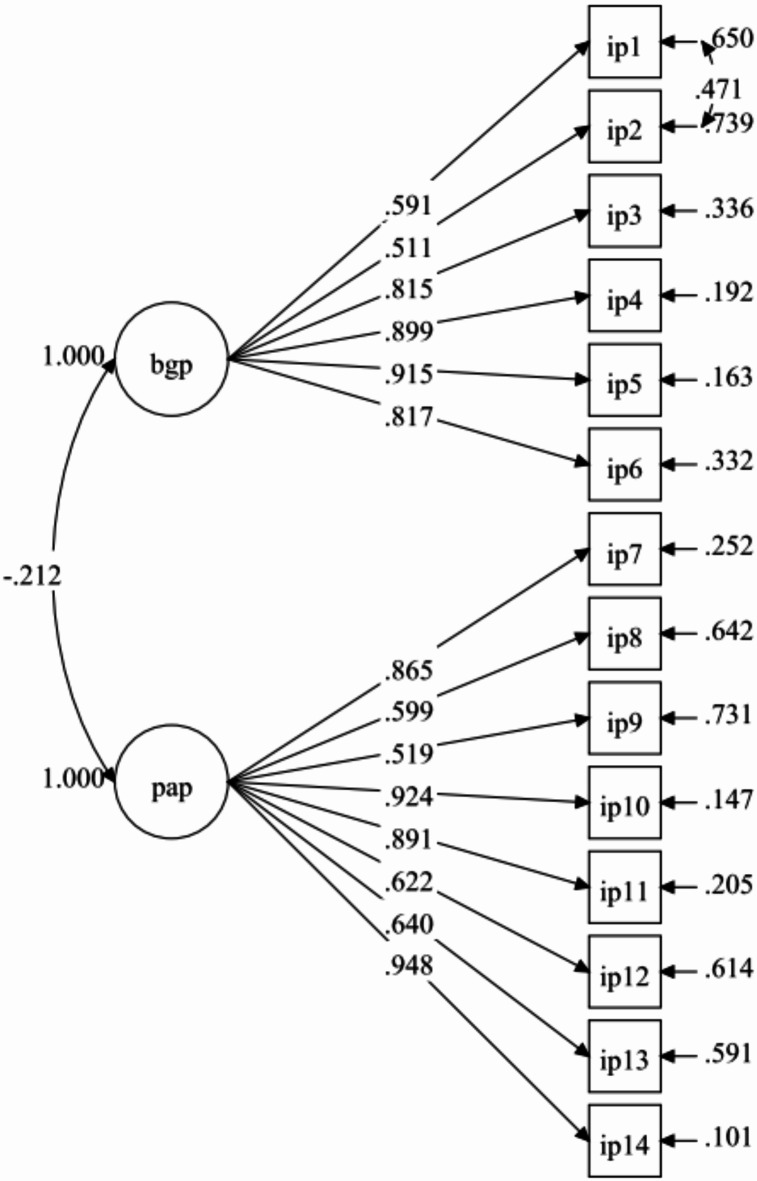
IP-Q measurement (Model-2).

Researchers further tested the EFA measurement model, which included 14 items reflecting two factors—biologically given potential (6 items) and personally acquired potential (8 items)—using CFA with an independent sample of 430 students. Results from Model-1 demonstrate that the fit indices were unsatisfactory (Table 3). All items, however, had standardized factor loadings higher than 0.40 (Fig. 2). The model fit indices improved (Fig. 3) when one pair of error covariances between items that belonged to the same factor were included. The model retained all items, and the fit indices for Model-2 were satisfactory (Table 3). The final model’s (Model-2) results showed factor loadings that were considered to be moderate to very good, with a range of 0.511 to 0.948 (Fig. 3).

**Table 3:** Summary for IP-Q Model fit indices (n = 430).

Path model	RMSEA (90% CI)	CFI	TLI	SRMR	RMSEA *p*-value
Model-1	0.078 (0.068, 0.088)	0.941	0.929	0.045	< 0.001
Model-2	0.060 (0.049, 0.070)	0.966	0.958	0.036	0.064

Model-2 with two correlated items residual: IP2 with IP1.

### Composite reliability (CR), average variance extracted (AVE), and discriminant validity

Table 4 presents the CR, AVE, and discriminant validity of the IP-Q. The CRs for the IP-Q factors were 0.878 for biologically given potential and 0.909 for personally acquired potential. The AVEs were 0.626 for biologically given potential and 0.590 for personally acquired potential. Thus, the IP-Q exhibited sufficient convergent validity^38^. The correlation coefficient between the factors was less than 0.85. Additionally, the squared correlation coefficients between the factors is lower than the AVE values. As such, the IP-Q has adequate discriminant validity^33,35^.

**Table 4:** Composite reliability (CR), average variance extraction (AVE), factor correlation and squared correlation for IP-Q final model.

Construct	CR (95% CI)	AVE	1	2	r^2^
BGB	0.878 (0.851, 0.906)	0.626	1	− 0.065	0.004
PAP	0.909 (0.897, 0.922)	0.590		1	

BGB = biologically given potential, PAP = personally acquired potential.

### Test–retest reliability

A total of 70 respondents completed the IP-Q twice over the course of seven days. For biologically given potential, the mean was 10.43 (SD = 5.06) on day 1 and 10.14 (SD = 4.94) on day 7, with an ICC value of 0.976 (95% CI: 0.962, 0.985). The mean was 21.36 (SD = 2.81) on day 1 and 20.59 (SD = 2.62) on day 7, with an ICC value of 0.953 (95% CI: 0.925, 0.970) for personally acquired potential.

## Discussion

Potentialities that stem from both biological inheritance and individual cultivation are not delineated by distinctions between body and mind. Numerous aspects of personally developed potential also manifest within the body, despite the presence of biologically endowed potential reflected in one’s physical makeup^1^. Individuals who engaged in physical activity during childhood tend to possess more active musculoskeletal systems compared to those who predominantly dedicated their youth to reading or computer activities. Anatomical and physiological variances illustrate disparities in personally acquired potentials, as demonstrated in this and various other instances^1,3^. We conducted a cross-sectional survey with 730 undergraduate students to develop a concise self-report tool for evaluating subjective individual potentials (IP-Q). The Meikirch model^1,3^ inspired the design of IP-Q, which incorporates two key factors: biologically given potential and personally acquired potential. Researchers anticipate that this tool will contribute to efforts to improve health outcomes and address the complex interplay of factors influencing holistic health care.

The IP-Q was assessed using four rating options, ranging from 1 (none) to 4 (severe) for biologically given potential and from 1 (not at all) to 4 (very often) for personally acquired potential. The scoring range for biologically given potential falls between 6 (minimum) and 24 (maximum), while for personally acquired potential, it ranges from 8 (minimum) to 32 (maximum). Lower scores in the biologically given potential domain indicate a perception of satisfactory health status, whereas higher scores indicate a perception of inadequate health. Conversely, in the personally acquired potential domain, lower scores suggest a lower sense of coherence, while higher scores indicate a higher sense of coherence. To compute the total score for this domain, reverse code items 8, 9, 11, 12, and 13, which are scaled in the negative direction.

The results of the content validity assessment reveal that both the I-CVIs and S-CVIs fall within the range of 0.83 to 1. Similarly, the face validity assessment yielded I-FVIs and S-FVIs of 1. These findings confirm satisfactory content validity and face validity^24,27,28^. The study employed two distinct samples of undergraduate students, predominantly adolescents, to examine the psychometric properties of the IP-Q, employing EFA with 300 participants and CFA with 430 participants. The economic progress of nations and the overall welfare of their populations rely on the health of adolescents^40^. This is especially important because there is a significant correlation between health and health-related behaviors during adolescence and into adulthood^40^. The transition from adolescence to adulthood also holds significant implications for individuals’ well-being and quality of life. Moreover, these developmental transitions are shaped by the economic and environmental circumstances prevailing in each country^41^.

Two factors were identified in the EFA analysis, with all 14 items retaining satisfactory factor loadings (above 0.50) on their respective constructs (KMO = 0.905; *p*-value < 0.001). Researchers further tested the EFA model using the CFA. The final model showed adequate fit indices, and all the items had sufficient factor loading on their respective constructs (0.456–0.938). The two constructs demonstrated acceptable internal consistency (> 0.6)^26^, composite reliability (> 0.70), and discriminant validity (< 0.85)^33,35^. Overall, the results confirm that the IP-Q possesses acceptable psychometric properties and can effectively assess an individual’s potential. With careful consideration of relevant theory, we incorporated two pairs of error covariances into the final model (one for each construct). Based on MI values obtained from the Mplus output, we added these residual covariances. Residual covariances are considered acceptable in social psychology when they have significance^42^.

The biologically given potentials consist of six items designed to assess an individual’s present health status and its potential impact on their daily functioning. When individuals evaluate their own health, they rely on information that holds significant predictive value^43^. Previous research findings indicate that self-perceived health remains as a reliable predictor of various outcomes, including the likelihood of developing chronic diseases^44^, recovering from illnesses^45^, experiencing functional decline^46^, and utilizing medical services^47^. This holds true even when considering more objective health indicators^47,48^. According to Bircher^1^, biologically given potential begins to decline shortly after birth and eventually reaches zero at the time of death. All somatic disorder, injury, or anomaly diminishes our biologically given potentials, either temporarily or permanently.

The personally acquired potential items comprise eight elements aimed at evaluating an individual’s ability to cope with challenges across past, present, and future contexts. These items encompass various facets of an individual’s physical, intellectual, and social resources. While the advancement of personal potential may decelerate in adulthood, it remains capable of growth as long as individuals are motivated to actively foster their development and reside in a social environment supportive of their well-being^1^. Cultivating positive emotions can enhance well-being and extend one’s lifespan^21,22^. Additionally, Antonovsky^20^ view suggests that individuals with a heightened sense of coherence often perceive their circumstances as manageable, meaningful, and comprehensible.

The present study does have certain limitations. Firstly, since the survey was conducted solely at one university, inferences regarding the study findings should be made with caution. However, the large sample size may give the study’s conclusions and results greater weight. Future studies should consider testing the measurement and structural invariance of the IP-Q across different cultures. Secondly, relying on a self-reported survey introduces the possibility of response bias, which may compromise the accuracy of the collected data. Participants were assured of the confidentiality of their information and instructed to provide truthful and accurate responses while refraining from discussing the survey with their colleagues. Thirdly, the study employed a convenience sampling method to select participants, which inherently limits the generalizability of the study findings. We recommend future studies employ a probability sampling approach such as simple random sampling or stratified random sampling to enhance the generalizability of the IP-Q. Finally, given the intricate nature of the study’s constructs, researchers may have omitted certain important items. Nevertheless, the study aims to develop a brief measure for assessing subjective individual potential.

## Conclusions

The present study develops a concise self-report instrument, the IP-Q, to assess biologically given and personally acquired potential. Item generation was guided by thorough literature reviews, along with assessments of content validity and face validity. Additionally, the study examines the construct validity and stability of the IP-Q. Findings suggest that the IP-Q demonstrates favorable psychometric properties within a sample of undergraduate students. This instrument is expected to play a crucial role in improving health outcomes and addressing the complex interplay of factors that influence holistic healthcare.

## Data Availability

The dataset supporting the findings of this article is available from the corresponding author on request.
